# Supplementary material for: Identification of photoperiod-induced specific miRNAs in the adrenal glands of Sunite sheep (Ovis aries)
Source: Front Vet Sci. 2022 Jul 22;9:888207. doi: 10.3389/fvets.2022.888207 (PMC9354845; doi:10.3389/fvets.2022.888207)
Supplement: Supplementary file 1 [file Data_Sheet_1.ZIP › Supplementary file/miRDeep2 file.zip!/miRDeep2 file/Know_Structure/SPLP42A3/oar-let-7f.pdf]

miRBase precursor : oar-let-7f
Total read count : 452905
oar-let-7f read count : 452861
remaining reads : 44

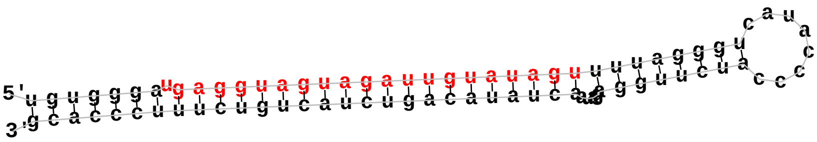

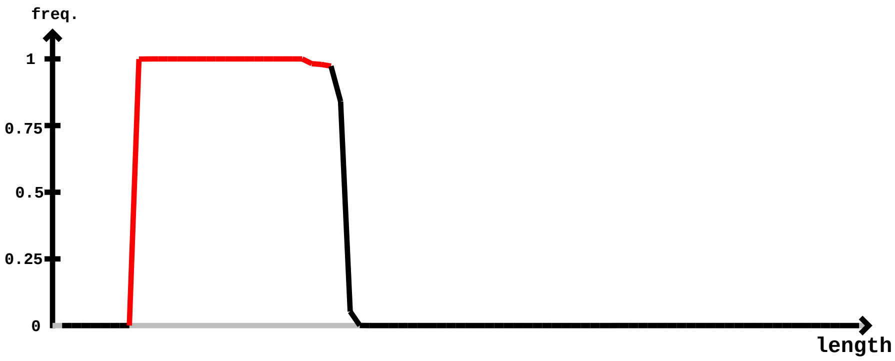


```
      oar-let-7f
5'- ugugggaugagguaguagauuguauaguuuuagggucauaccccaucuuggagauaacuauacagucuacugucuuucccacg   -3'   exp
    .((((((.((((((((((((((((((((((((((((........))))))))....)))))))))))))))))))))))))).    reads    mm    sample
    ......augagguaguagauuguau..........................................................    1        0     seq
    ......augagguaguagauuguauagu.......................................................    6        0     seq
    ......augagguaguagauuguauaguu......................................................    26       0     seq
    ......Gugagguaguagauuguauaguu......................................................    2        1     seq
    ......augagguaguagauuguauaguuu.....................................................    3        0     seq
    .......ugagAuaguagauugua...........................................................    1        1     seq
    .......ugagguaguagauugua...........................................................    44       0     seq
    .......ugagguaguagauuguU...........................................................    4        1     seq
    .......ugagguaguUgauuguau..........................................................    3        1     seq
    .......ugaAguaguagauuguau..........................................................    62       1     seq
    .......ugagguaguagauuguau..........................................................    7954     0     seq
    .......ugagguaguagauuguUu..........................................................    2        1     seq
    .......ugagguaguagauuguaua.........................................................    1350     0     seq
    .......ugagguaguagauuguauag........................................................    2773     0     seq
    .......ugagguaguagauugAauag........................................................    1        1     seq
    .......ugagguaguagauuguauagu.......................................................    60070    0     seq
    .......ugagguaguagauugAauagu.......................................................    7        1     seq
    .......ugagguaguagauuguauaguu......................................................    356547   0     seq
    .......ugagguaguagauuguauaguuu.....................................................    22968    0     seq
    .......ugagguaguagauuguauaguuG.....................................................    587      1     seq
    .......ugagguaguagauuguauaguuGu....................................................    27       1     seq
    .......ugagguaguagauuguauaguuuu....................................................    124      0     seq
    .......ugagguaguagauuguauaguuuua...................................................    7        0     seq
    ........gagguaguagauuguau..........................................................    4        0     seq
    ........gagguaguagauuguauag........................................................    1        0     seq
    ........gagguaguagauuguauagu.......................................................    30       0     seq
    ........gagguaguagauuguauaguu......................................................    205      0     seq
    ........gagguaguagauuguauaguuG.....................................................    1        1     seq
    ........gagguaguagauuguauaguuu.....................................................    11       0     seq
    .........agguaguagauuguauagu.......................................................    1        0     seq
    .........agguaguagauuguauaguu......................................................    6        0     seq
    .........agguaguagauuguauaguuG.....................................................    1        1     seq
    .........agguaguagauuguauaguuGu....................................................    25       1     seq
    ..........gguaguagauuguauaguu......................................................    4        0     seq
    ..........gguaguagauuguauaguuu.....................................................    1        0     seq
```

oar-let-7f

```
uguggga**ugagguaguagauuguauagu**uuuagggucauaccccaucuuggagauaacuauacagucuacugucuuucccacg
...........guaguagauuguauaguu.....................................................    1    0    seq
............uaguagauuguauaguC.....................................................    1    1    seq
.............................uuagggucauaccccaucuugga..............................    1    0    seq
.............................uuagggucauaccccaucuuggaga............................    1    0    seq
.........................................................cuauacagucuacugucu.......    1    0    seq
.........................................................cuauacagucuacugucuuu.....    2    0    seq
.........................................................cuauacagucuacugucuuuc....    9    0    seq
.........................................................cuauacagucuacugucuuucc...   30    0    seq
```
